# Impact of Active Esophageal Cooling on Fluoroscopy Usage During Left Atrial Ablation

**DOI:** 10.19102/icrm.2021.121101

**Published:** 2021-11-15

**Authors:** Jason Zagrodzky, Shane Bailey, Shailee Shah, Erik Kulstad

**Affiliations:** ^1^Department of Electrophysiology, St. David’s South Austin Medical Center, Austin, TX, USA; ^2^Atune Medical, Chicago, IL, USA; ^3^Department of Emergency Medicine, UT Southwestern Medical Center, Dallas, TX, USA

**Keywords:** Atrial fibrillation, esophageal cooling, esophageal injury, fluoroscopy, radiofrequency ablation

## Abstract

Risks to collateral structures exist with radiofrequency (RF) ablation of the left atrium to obtain pulmonary vein isolation (PVI) for the treatment of atrial fibrillation. Passive luminal esophageal temperature (LET) monitoring is commonly utilized, but increasing data suggest limited benefits with LET monitoring. In contrast, active cooling of the esophagus has been shown to significantly reduce esophageal injury. Active cooling of the esophagus also avoids the need for stopping and repositioning an LET probe during use, which may reduce the need for fluoroscopy use. This study aimed to measure the impact on fluoroscopy use during RF ablation with esophageal cooling using a dedicated cooling device in a low-fluoroscopy practice. All patients who underwent PVI over a one-year timeframe by a single provider were analyzed. Patients undergoing PVI prior to the incorporation of an esophageal cooling protocol into standard ablation practice were treated with traditional LET monitoring. Patients treated after this point received active esophageal cooling, in which no LET monitoring is utilized. A total of 280 patients were treated; 91 patients were treated using LET monitoring, and 189 patients were treated with esophageal cooling. The mean total fluoroscopy time before the implementation of the esophageal cooling protocol in 91 patients was 194 seconds [standard deviation (SD): 182 seconds] per case, with a median of 144 seconds. The mean total fluoroscopy time after implementation in 189 patients was 126 seconds (SD: 120 seconds) per case with a median of 96 seconds, representing a reduction of 35% per case (p < 0.0001, Mann–Whitney U test). In this largest study to date of active esophageal cooling during PVI, a 35% reduction in fluoroscopy time compared with patients who received LET monitoring was found. This reduction was seen despite an already low fluoroscopy usage rate in place.

## Introduction

Ablation of the left atrium to attain pulmonary vein isolation (PVI) is being increasingly utilized for the treatment of atrial fibrillation (AF).^1^ PVI using radiofrequency (RF) energy or cryothermal energy risks collateral damage to surrounding structures, particularly to the esophagus, with atrioesophageal fistula (AEF) being the most extreme and lethal example.^2–6^ A number of approaches to reducing the risk of AEF exist, including reducing power at the posterior wall, monitoring luminal esophageal temperature (LET), deviating the esophagus during ablation, and cooling or warming the esophagus.^7–12^ Most methods have not shown benefits, and recent randomized controlled trials (RCTs) have either shown no benefit or trends toward harm with LET monitoring.^13–19^ On the other hand, two pilot RCTs suggested benefits with active cooling, and these benefits have been confirmed in a larger RCT, which demonstrated an 83% reduction in endoscopically identified esophageal lesions using active cooling.^20–22^ Demonstration of a reduction in AEF formation using active cooling has not yet been confirmed; however, significant data exist to suggest that high-grade esophageal lesions progress to fistulae at a rate of approximately 9.6%, while low-grade lesions do not in general progress to fistulae.^23^ In addition, new data recently presented have shown no adverse events or fistula development after 3,200 cases of active esophageal cooling to date.^24^

Actively cooling the esophagus during RF ablation has been reported to have several additional benefits, including improved procedural efficiency (allowing point-to-point ablation to occur without interruption from LET monitoring alarms), improved transmurality of lesions, and reduced fluoroscopy requirements.^11,12,25–29^ Reduction of fluoroscopy usage is of increasing interest, as career-long radiation exposure to clinicians can result in higher lifetime risks of cataracts, stroke, atherosclerosis, and brain cancer.^30^

**Figure 1:**
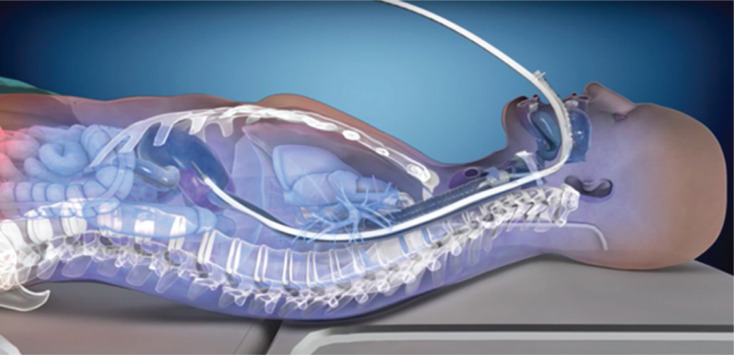
Esophageal temperature-management device in place during active esophageal cooling.

**Figure 2:**
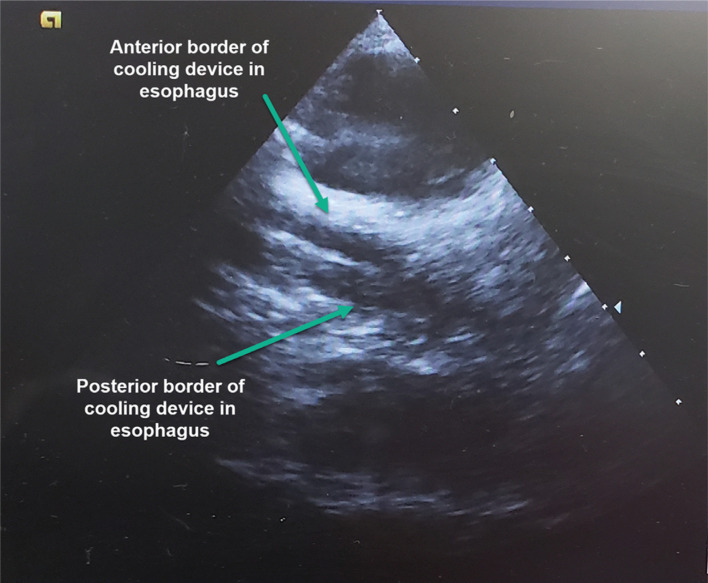
Visualization of commercially available esophageal cooling device on ICE.

A commercially available device **(Figure 1)** has been cleared by the United States Food and Drug Administration for patient temperature management via the esophagus and is increasingly being used in the electrophysiology (EP) lab. The device is placed into the esophagus and provides a closed-circuit flow of water at a temperature setpoint chosen by the operator to provide cooling or warming. Once placed, no further manipulation of position is required, and visualization of the device is possible on intracardiac echocardiography (ICE) **(Figure 2)**, enabling significant reduction, or even elimination, of the need for the use of fluoroscopy. This study aimed to measure the impact of active esophageal cooling on the use of fluoroscopy during RF ablation by comparing fluoroscopy requirements before and after the implementation of active esophageal cooling.

## Methods

This study was a retrospective review under Institutional Review Board approval of all patients with AF who were treated with left atrial RF ablation from October 2018 to October 2019. In order to minimize variance between providers and avoid the influence of different control conditions used by different providers, the analysis focused on the cases performed by a single experienced electrophysiologist who primarily performs wide-area circumferential pulmonary vein ablation and posterior wall isolation procedures. The posterior wall was isolated using a combination of roof and floor linear lesions along with additional lesions as needed to complete the isolation. Patients were given general anesthesia for the ablation procedure. Anticoagulation was administered prior to the ablation in a standard manner, with patients heparinized to a target activated clotting time of 350 seconds. In the left femoral vein, a steerable decapolar catheter (Webster CS Catheter; Biosense Webster, Inc., Diamond Bar, CA, USA) was placed in the coronary sinus for pacing and recording, and an ICE catheter (AccuNav; Siemens, Mountain View, CA, USA) was positioned in the right atrium to guide the transseptal puncture. In the right femoral vein, two transseptal sheaths were positioned in the left atrium. A circular mapping catheter (PentaRay or Lasso; Biosense Webster, Inc.) was utilized to obtain electroanatomical maps, and a three-dimensional geometry was created using the Carto system (Biosense Webster, Inc.). For ablation, an externally irrigated ablation catheter (ST/SF™; Biosense Webster, Inc.) was used in all cases. The pulmonary veins were isolated by delivery of RF applications circumferentially to the antral regions to produce a minimum of entrance and exit block for at least 20 minutes, confirmed during isoproterenol infusion at 10 μg/min. A Stockert™ generator (Biosense Webster, Inc.) was used to deliver RF energy, with a setpoint of 40 W on all patients and all areas of the left atrium. The Surepoint measure (Biosense Webster, Inc.) was utilized during ablations, with a target of 350 units on the posterior wall and 450 units on the anterior wall, lateral wall, and septum. Catheter tip temperature, power, and impedance were recorded for each RF energy application.

Prior to implementing the use of active cooling, standard LET monitoring was utilized. In LET-monitored patients, the majority of sensors used were single-sensor probes, with approximately 10% of them receiving a multi-sensor probe (Circa S-Cath™; Circa Scientific, Inc., Englewood, CO, USA). Energy delivery was discontinued when the maximum LET on the single-sensor temperature probe or on any sensor of the multi-sensor probe rose by more than 0.2°C/s, or exceeded 39°C. In patients treated with active cooling, the position of lesions on the posterior wall was adjusted if needed to avoid ablation directly over the esophagus if the tissue thickness (atrium and esophageal wall) was less than approximately 2 mm as viewed on ICE. Otherwise, the ablation proceeded in a point-to-point fashion uninterrupted by pauses or alarms.

Total fluoroscopy time was determined from the formal procedure record obtained for each patient from the EP lab. Except for the change to the esophageal cooling protocol in the treatment group, the RF ablation procedure for patients in both groups was the same. Fluoroscopy times for each patient were analyzed using SPSS Statistics version 26 (IBM Corporation, Armonk, NY, USA), with descriptive statistics [mean, median, and standard deviation (SD) values] and comparisons between groups using the Mann–Whitney U test reported.

## Results

A total of 280 patients were treated over the one-year time frame; 91 patients were treated with RF ablation in the five-month period prior to the transition to the use of an active esophageal cooling protocol. These patients received no esophageal cooling but had LET monitored with single-sensor temperature probes. The mean total fluoroscopy time in this group was 194 seconds (SD: 182 seconds) with a median of 144 seconds **(Table 1)**.

**Figure 3:**
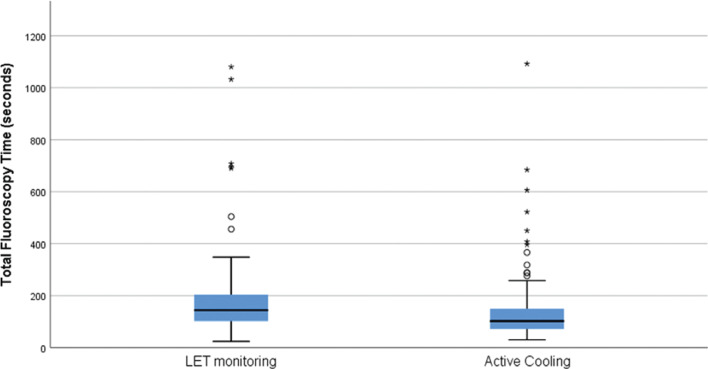
Boxplots of total fluoroscopy time (s) for the two groups compared. LET: luminal esophageal temperature.

**Figure 4:**
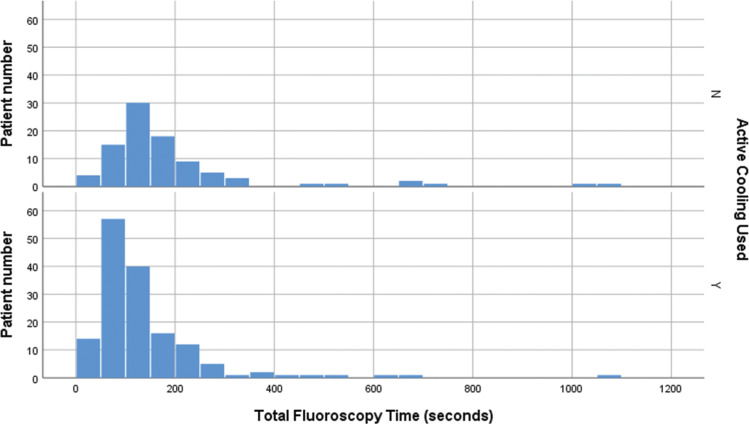
Histograms of total fluoroscopy time (s) for the two groups compared.

A total of 189 patients were treated with RF ablation in the eight-month period after the implementation of active esophageal cooling. The total fluoroscopy time after the implementation of the protocol was 126 seconds (SD: 120 seconds) with a median of 96 seconds. Comparing groups, a reduction in fluoroscopy time of 35% was found (p < 0.0001, Mann–Whitney U test). **Figure 3** compares boxplots of total fluoroscopy time for the two groups, and **Figure 4** compares histograms of total fluoroscopy time for the two groups. Overall procedure times were not significantly different between groups, with first sheath placement to the completion of the procedure averaging 69.6 minutes (SD: 21 minutes) before the use of active esophageal cooling and 72.7 minutes (SD: 17 minutes) after the implementation of active esophageal cooling (p = 0.22), respectively.

## Discussion

In this review of 280 patients, the use of active esophageal cooling was associated with a reduction in fluoroscopy usage when compared to traditional LET monitoring. Although the retrospective nature of this study does not provide a means to definitively demonstrate reasons for this reduction, the reduction in fluoroscopy time is likely a consequence of eliminating the need for repositioning of the temperature probe typically used for LET monitoring during ablation. Esophageal temperature monitoring with LET probes often requires repositioning single-sensor probes to ensure their optimal positioning opposite the point of contact with the RF catheter against the posterior wall of the atrium, and suboptimal positioning risks missing the detection of a rise in temperature. A recent editorial commenting on LET rise noted that good practices for esophageal temperature monitoring involve repeatedly adjusting the single thermistor temperature probe position to ensure its proximity to the ablation catheter during continuous data collection.^31,32^ Multi-sensor probes may involve less repositioning but still require fluoroscopy for positioning. With active esophageal cooling, the location of the cooling device is confirmed at the initial placement and can be confirmed with ICE alone, ensuring complete coverage of the esophagus and eliminating the need for readjustment or repositioning of the device during ablation. This is a device generally placed by anesthesia, without the need for involvement by the electrophysiologist. As such, there is no relevant learning curve, and the ablation itself proceeds as usual, without the need for learning any new techniques or reacting to temperature readouts, temperature alarms, or other interruptions.

Currently utilized approaches to esophageal protection have different requirements for fluoroscopy during use, with some, such as deviation and passive temperature monitoring, requiring repositioning or active manipulation under fluoroscopy during ablation. Given the increased efforts to reduce fluoroscopy time to as low as practicable, and the increased awareness of risks associated with radiation exposure to patients and medical staff, any reduction in fluoroscopy time is desirable.^30,33–38^ Assuming an average per-procedure effective dose of 15 mSv and an annual provider exposure of 5 mSv, any per-procedure reduction in radiation exposure can benefit both patients and providers.^39^

The importance of addressing preventable complications has been highlighted in recent data suggesting higher real-world mortality and complication rates with PVI than has been reported in clinical trials.^40^ Esophageal injury remains a concern during atrial ablation, and numerous approaches have been utilized or developed in an attempt to reduce or eliminate the potential for esophageal injury, which can result in the development of AEF.^7–12^ The use of high-power, short-duration ablation and the development of an ablation index may offer further safety improvements, but benefits to date in reducing esophageal injury in formal studies using objective endpoints (either magnetic resonance imaging– or endoscopy-proven esophageal injury) remain unclear.^41,42^ On the other hand, currently available technology that provides active esophageal cooling has suggested benefits in multiple pre-clinical and clinical studies,^11,12,20–22,43,44^ with two pilot RCTs and one confirmatory RCT demonstrating significant reductions in esophageal injury.^20–22^ Procedural efficiency improvements in the form of reduced fluoroscopy requirements may add another benefit to this approach.^43,45,46^

With the low baseline fluoroscopy time encountered in the practice studied here, a 35% reduction translates into a rather low absolute reduction of fluoroscopy usage. Nevertheless, even with only a one-minute reduction per case, when multiplied out over the number of cases of left atrial ablation increasingly being performed, the total reduction for a given operator over time (eg, a reduction of 5 mSv/year) can translate into a considerable and possibly clinically meaningful reduction in fluoroscopy exposure over a lifetime of practice.^33–37,39,47^ With increasing emphasis on reducing fluoroscopy exposure and the recommendations that EP labs worldwide should heed the as low as reasonably achievable principle, further reduction of radiation exposure to both patients and procedural staff is increasingly being sought, and this may in turn be of interest to operators and laboratory staff in general.^7^ The additional downsides of fluoroscopy usage (the wearing of lead for all of the laboratory staff) may not be impacted by a percentage reduction, but the fact that this approach can also be utilized in zero-fluoroscopy settings is also likely to be of interest to operators generally.

Total procedure times were not found to be markedly different in our measurements. This is perhaps due to the fact that total procedure times were likewise quite low to begin with, but also may be due to the practice pattern of the operator, where the use of fluoroscopy for repositioning a temperature sensor added a larger amount of time as a percentage of the total fluoroscopy use than it does in total procedure times. In analyses of other practices, greater time reductions have been seen, including some unpublished reports of savings of 30 minutes or more, but these data appear to be very dependent on practice styles (ie, temperature thresholds used for cessation of energy deposition, type of temperature sensor utilized, etc.).^48^

The risks from the device studied here have been shown to be extremely low, with recently presented data showing no adverse events, fistulae, or other complications in 3,200 cases.^24^ The costs are similar to—or, in some cases, much less than—other advanced technologies utilized for esophageal protection, so on balance, the cost–benefit analysis appears to be reasonable. Many practitioners utilize single-sensor temperature probes with mapping catheters sutured or taped adjacently in order to visualize the temperature sensor on the atrial map; as the cooling device does not require repositioning, and the device is visible on ICE, the use of an adjacent mapping catheter is obviated, further reducing the cost of this approach. The additional data emerging on the impact of the device include lower long-term AF recurrence rates after ablation (presumably due to the facilitation of point-to-point ablation without interruption from overheating alarms) and lower incidences of post-ablation pericarditis and gastroparesis, and these appear to offer yet further advantages not described in this current analysis, but that may weigh further on the benefit side of the use case analysis.^20,24^ Because the device is generally placed by an anesthesiologist or a certified registered nurse anesthetist (CRNA) and not by the electrophysiologist, there is no relevant learning curve nor a change to the ablation procedure itself. The learning curve for anesthesiologists or CRNAs to place the device is minimal, as placement is similar to that of a standard orogastric tube.

### Limitations

This study relied on a retrospective review of data collected by the EP lab in accordance with the standard procedure and, as such, cannot eliminate potential unmeasured confounders or other sources of bias in the manner of a formal RCT. Nevertheless, as no other substantial changes were made in ablation procedures or patient care pathways during the study period, a substantial confounder appears unlikely. Moreover, the influence of variations in treatment by the provider was reduced by restricting the analysis to a single provider and including all patients treated by the same provider, yielding a large dataset of 280 patients to analyze. The baseline fluoroscopy time was low, and further study will be required to determine if similar reductions will be seen in higher utilizers of fluoroscopy. These prospective studies are underway (NCT04087122 and NCT04063761). LET monitoring was via single-sensor temperature probes in most cases and multi-sensor probes in a smaller percentage, so these results may not be generalizable to practices primarily utilizing multi-sensor temperature probes.

## Conclusions

In this largest study to date of active esophageal cooling during PVI, a 35% reduction in fluoroscopy time compared with patients who received LET monitoring was found. This reduction was seen despite an already low fluoroscopy usage rate in place.

## Figures and Tables

**Table 1:** Summary of Statistics for Each Group

	Fluoroscopy Time (s)	
	LET Monitoring (n = 91)	Esophageal Cooling (n = 189)
Mean	194	126
Standard error of the mean	19	9
Median	144	96
Maximum	1,080	1,092
Minimum	24	0
95.0% lower CL for the mean	156	109
95.0% lower CL for the median	132	96
95.0% upper CL for the mean	232	143
95.0% upper CL for the median	180	108
Range	1,056	1,092

CL: confidence limit; LET: luminal esophageal temperature.

The LET monitoring group did not receive esophageal cooling, and the esophageal group did not undergo LET monitoring.
